# Apoptotic Induction by Biosynthesized Gold Nanoparticles Using *Phormidesmis communis* Strain AB_11_10 against Osteosarcoma Cancer

**DOI:** 10.3390/biomedicines12071570

**Published:** 2024-07-15

**Authors:** Reham Samir Hamida, Sahar M. AlMotwaa, Waad A. Al-Otaibi, Haifa A. Alqhtani, Mohamed Abdelaal Ali, Mashael Mohammed Bin-Meferij

**Affiliations:** 1 Institute for Protein Research, Osaka University, Osaka 565-0871, Japan; 2 Department of Chemistry, College of Science and Humanities, Shaqra University, Shaqra 11961, Saudi Arabia; 3 Department of Biology, College of Science, Princess Nourah Bint Abdulrahman University, P.O. Box 84428, Riyadh 11671, Saudi Arabia; 4 Plant Production Department, Arid Lands Cultivation Research Institute, City of Scientific Research and Technological Applications (SRTA-CITY) New Borg El-Arab, Alexandria 21934, Egypt

**Keywords:** biofabrication, metallic nanoparticles, cyanobacteria, flow cytometry

## Abstract

*Phormidesmis communis* strain AB_11_10 was isolated and identified using microscopy and 16s rRNA sequencing, and its phytochemical constituents were determined using liquid chromatography-quadrupole time-of-flight mass spectrometry. The isolate had a segmented filamentous shape with a blue-green color. Many biomolecules, including organic compounds, amino acids, and fatty acids, were detected. *P. communis* strain AB_11_10 was used to synthesize gold nanoparticles (Ph-AuNPs) by adjusting the optimum reaction conditions. The concentration, algal/precursor ratio, temperature, reaction time, and pH significantly influenced the synthesis of the Ph-AuNPs. Mixing 1 mL of 0.5 mM of HAuCl_4_ with 1 mL of algal extract and exposing the mixture to 100 °C for 30 min at pH 5.6 were the optimum conditions for the biosynthesis of Ph-AuNPs at a wavelength of 524.5 nm. The Ph-AuNPs were characterized using TEM, SEM, EDX, and mapping Zeta sizer and FTIR. The Ph-AuNPs had quasi-spherical to triangular shapes with an average diameter of 9.6 ± 4.3 nm. Ph-AuNPs composed of 76.10 ± 3.14% of Au and trace amounts of carbon and oxygen were detected, indicating that the *P. communis* strain AB_11_10 successfully synthesized Ph-AuNPs. The hydrodynamic diameter of the Ph-AuNPs was 28.5 nm, and their potential charge was −17.7 mV. O-H, N-H, C=C, N-O, C-H, and C-O were coated onto the surfaces of the Ph-AuNPs. These groups correspond to algal phytochemicals, which may have been the main reducing and stabilizing substances during the Ph-AuNP synthesis. The therapeutic activity of the Ph-AuNPs against osteosarcoma cancers was examined in MG-63 and SAOS-2 cell lines, while their biocompatibility was tested against Vero cell lines using a sulforhodamine B assay. The Ph-AuNPs had potent antitumor activity against the MG-63 and SAOS-2 cells, with a low toxicity toward Vero cells. Flow cytometry and cell cycle arrest analyses revealed that the Ph-AuNPs enhanced the apoptotic pathway and arrested the cell cycle in the MG-63 and SAOS-2 cells. *P. communis* strain AB_11_10 provides a new source to synthesize small, stable, and biocompatible AuNPs that act as apoptotic enhancers in osteosarcoma.

## 1. Introduction

Osteosarcoma (OS) is the most prevalent malignant bone tumor and primarily arises in the metaphyses of long bones; the majority of OS cases affect children and adolescents [1,2]. Lung metastases affect 15–20% of diagnosed OS patients, which correlates with its high mortality rate [3,4]. OS is characterized by an increased risk of recurrence, aggressive invasion, high metastasis, and low survival rates [5,6,7]. There are an estimated ~4.3 and ~3.4 OS cases per million in males and females, respectively [8]. The etiology of OS is multifaceted, including genetics, epidemiology, and environmental factors, and remains inadequately understood [9,10]. Neoadjuvant chemotherapy followed by surgery has been suggested as an OS therapy to improve survival rates [11]. Despite recent improvements, conventional therapeutic methods for OS lead to more toxic effects on healthy tissues, including widespread or non-targeted delivery, a rapid clearance rate, a high incidence of unwanted side effects, and drug resistance [12,13]. To circumvent these issues, the development of novel and selective therapeutic strategies is required to improve the survival and quality of life of patients with OS.

Nanotechnology is becoming a leading concept and practice in diverse fields of research, including agriculture, food, energy, and biomedicine [14]. The primary properties of nanoparticles (NPs) include a small size, a nanoscale range of 1–100 nm, a large surface area to surface volume ratio, and great optical, chemical, magnetic, and mechanical properties [15,16]. These properties are fundamental for forming NPs that are suitable for use in the biomedical field as antibiotics, anticancer, and antioxidant agents [15,17,18]. The multifunctional theranostic properties of the NPs of noble metals such as gold, silver, iron, and cobalt make them highly suitable for medical applications [19].

Among the different noble metal NPs, AuNPs have unique surface plasmon resonance properties, facile synthesis, tunable sizes, and have attracted considerable attention in various fields [20,21,22]. Furthermore, their multifaceted abilities have well-characterized properties such as versatility, biocompatibility, relative inertness, and general stability [20]. AuNPs enhance antitumor activity through cell cycle arrest and induced apoptosis [23]. AuNPs have been previously investigated in osteosarcoma cell lines (MG-63). AuNPs can promote DNA fragmentation, the expression of caspase 3, and enhance apoptosis via the generation of ROS and restriction of mitochondrial membrane potential [24]. AuNPs have been shown to upregulate the expression of pro-apoptotic Bax concurrently with the downregulation of Bcl-2 in MG-63 cell lines [25]. Rahim et al. showed that glycogenic AuNPs inhibit the growth of SAOS-2 cells, and the stained cells revealed apoptosis characteristics such as apoptotic bodies, chromatin condensing, and membrane blebbing [26]. Ghahremani et al. found that a combination of AuNPs with chemotherapy and microwave hyperthermia in the SAOS-2 cell line indicated synergistic effects and a decreased survival rate [27].

Despite the various methods for synthesizing AuNPs, including physical, chemical, and biological methods [28], NPs mediated by physical and chemical synthesis methods threaten living organisms and the environment because they generate harmful by-products and utilize high levels of toxic chemicals, heat, and energy [17,29,30]. To circumvent the challenges of the conventional destructive effects associated with the physical and chemical synthesis of NPs, an alternative approach known as the green synthesis of NPs has evolved [20]. Green synthesis, popularly known as “biological synthesis”, is eco-friendly, cost-effective, and entails facile NP synthesis methods [29,30]. Biological agents, including bacteria, microalgae, cyanobacteria, plants, and others, offer facile, cost-effective, and ecofriendly synthesis approaches for NPs that consume little energy [31,32]. These organisms reduce the metal precursors for NPs through various mechanisms such as bioaccumulation and enzyme reduction [33,34]. However, the exact mechanism for the synthesis of NPs using organisms is still undiscovered. The advantages of using biological entities such as cyanobacteria for AuNPs’ synthesis include: (I) ease of culture, (II) the ability to adapt to environmental temperatures and atmospheric pressure changes, (III) a remarkable growth rate, (IV) the biosorption capacity of heavy metal ions and various intracellular biocompounds, (V) their environmental impact, (VI) by adjusting the growth conditions or the organism used, it is possible to influence the size, shape, and dispersity of the produced nanoparticles, (VII) biogenic NPs are more biocompatible agents toward normal cells with a high selectivity toward cancer cells compared to NPs synthesized by conventional methods, and (VIII) biogenic NPs have a great stability in salt-containing solutions compared to other NPs synthesized by conventional methods [34,35,36].

Several studies have revealed that various cyanobacterial strains can form various NPs, including metallic NPs, such as AgNPs, AuNPs, and metal oxide NPs [35,37,38,39,40]. Moreover, the cyanobacterial-assisted synthesis of NPs offers potent photocatalytic, anticancer, antibacterial, antifungal, and antialgal activities [35]. *Phormidium commune* strain *AB_11_10* is a new cyanobacteria species with little information about its morphology, metabolite compositions, and no data about its potential to synthesize NPs. Thus, our study focuses on filling this gap in knowledge by reporting new findings about its phytochemical compositions and reducing the potential of *Phormidium commune* strain *AB_11_10* to synthesize AuNPs. Therefore, the novelty of the present study lies in its ability to isolate and identify *P. communis* strain AB_11_10 and use its cell biomass phytochemicals to interact with HAuCl_4_ to synthesize small Ph-AuNPs by optimizing the synthesis reaction conditions. Various physicochemical techniques have been used to characterize biogenic Ph-AuNPs. Additionally, our Ph-AuNPs have a high stability, biocompatibility, and therapeutic efficacy against osteosarcoma cancer as an alternative to traditional chemically synthesized AuNPs.

## 2. Experimental Section

### 2.1. Cyanobacteria Isolation and Identification

Cyanobacteria were collected from soil in Borg Al-Arab, Alexandria, Egypt, kept in sterile containers, and cultured in Petri dishes containing BG11 medium for one week. The Petri dishes were kept in an incubator and exposed to light (2000 ± 200 lx) for 12 h and dark for 12 h. To obtain purified colonies of the cyanobacteria, isolation and purification were performed using serial dilution and BG11 agar plates, according to methods mentioned previously in our paper [41] and based on Bolch et al. [42]. The morphology of the purified colonies was examined using light and scanning electron microscopy (SEM). Before the SEM investigation, an aliquot (1 mL) of algal suspension was centrifuged at 4700 rpm for 10 min, washed three times, and fixed in 70% ethanol. Small pieces of algal mats were attached to a sterilized piece of glass, taped to a copper stub, dried, and coated using a platinum coater (JEC-3000FC, Jeol, Tokyo, Japan) at 15 kV. To identify the samples genetically, genomic DNA was extracted according to the manufacturer’s instructions (Attogene, Austin, TX, USA). Amplification of the purified DNA was performed by PCR using forward and reverse primers. The amplified DNA was sent for 16s rRNA sequencing (Macrogen, Seoul, Republic of Korea).

### 2.2. Algal Extract

The reducing and stabilizing molecules required to fabricate the HAuCl_4_ precursor into the Ph-AuNPs were obtained by centrifuging the algal culture after 15 d of their growth cycle at 4700 rpm for 10 min, washing the algae three times, drying for 1 d using a freeze-drying machine, mixing 200 mg of algal powder with 100 mL of distilled (dist.) H_2_O, and keeping it in a water bath at 60 °C for 30 min. The mixture was then allowed to cool to ambient temperature and was centrifuged, and the supernatant was filtered through Whatman filter paper No. 1. The filtrate was stored in a sterilized Falcon tube until further use.

### 2.3. Chemical Composition Using Liquid Chromatography Quadrupole Time-of-Flight Mass Spectrometry (LC-QTOF-MS)

LC-QTOF analysis of the algal biomass extracts was performed using an X500R LC-QTOF instrument (SCIEX, Framingham, MA, USA) equipped with an Inertsustan C18 column (25 cm × 4.6 mm × 5 μm; mass range TOF: up to 40 kDa; precursor ion selection: 5–2250 *m*/*z*; mass accuracy over time: <2 ppm root mean square error for 12 h; LC-MS ionization sources: turbo V ion source with twin sprayer ESI probe and twin sprayer atmospheric pressure chemical ionization probe; TOF-MS resolution and speed: ≥42,000 (full width at half maximum) measured on a (M+6H)^6+^ charge isotope cluster for bovine insulin at *m*/*z* 956). The mobile phase consisted of 0.1% formic acid in water (eluent A: a mixture of formic acid and water at a volume ratio of 1:1000) and acetonitrile (eluent B). The gradient program was as follows: 97–3% B (0 min), 97–3% B (0–5 min), 10–90% B (5–18 min), 10–90% B (18–23 min), 97–3% B (23–27 min), and 97–1% B (27–30 min). A volume of 6 μL was injected for each standard or sample, and the flow rate was set at 1 mL/min. Nitrogen gas nebulization was set at 50 psi, while curtain gas was 30 psi, and the temperature was set to 500 °C. The ion spray voltage was set to 5000 V. A complete mass scan ranging from 50 to 1000 *m*/*z* was used, and MS/MS analyses were performed in automatic mode with a collision energy of 10 V for fragmentation. Peaks were identified in both the positive and negative modes, and instrument control, data acquisition, and processing were performed using SCIEX OS 2.0.1 software (SCIEX, MA, USA).

### 2.4. Optimization of Ph-AuNP Synthesis and Physicochemical Characterizations

**Scheme 1:**
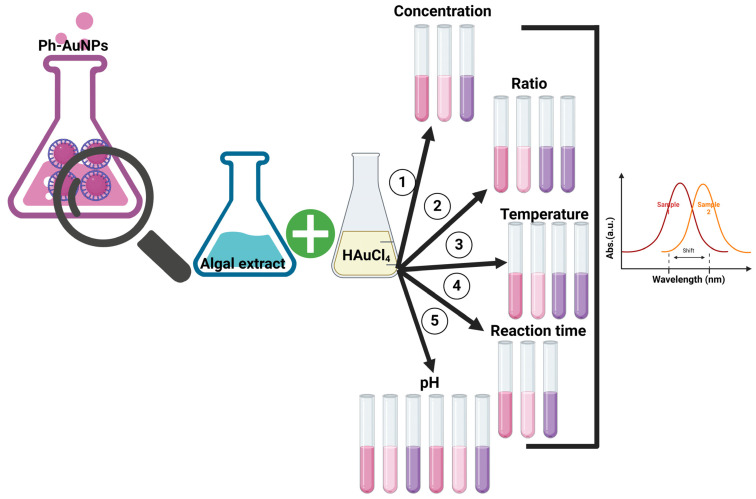
Illustrating the optimization process of synthesizing Ph-AuNPs using *Phormidesmis communis* strain AB_11_10.

Optimizing the parameters of the NP synthesis process, such as the precursor concentration, algal-to-precursor ratio, temperature, reaction time, and pH, was performed, resulting in the formation of small, stabilized NPs. To achieve this, we mixed 1 mL of algal extract with 1 mL of different concentrations of HAuCl_4_ (0.5, 1, and 2 mM) under constant conditions, including exposure to 80 °C for 30 min without adjusting the reaction pH. After incubation, the wavelengths of an aliquot of each Ph-AuNP reaction were measured using UV-spectroscopy (Model UV-1800, Shimadzu, Kyoto, Japan). Subsequently, 1 mL of the algal extract was mixed with different precursor volumes at (*v*/*v*) ratios (1:1, 1:2, 1:4, and 1:9) at the optimum concentration (0.5 mM) of HAuCl_4_ under other constant parameters. To investigate the role of temperature in the synthesis process of the Ph-AuNPs, 1 mL of algal extract was mixed with 1 mL of 0.5 mM HAuCl_4_ and maintained in a water bath at different temperatures (40, 60, 80, and 100 °C) for 30 min without adjusting the pH of the reaction. Under optimum conditions, the reaction time was assessed by treating the reactions at 100 °C for 15, 30, and 60 min. The influence of pH was investigated by varying the pH of the reactions of the algal extracts and HAuCl_4_ using 0.1 M NaOH or 0.1 M HCL to be 6, 7, 8, 10, and 12 under other optimum conditions. The absorption peaks of the Ph-AuNP reactions guided the selection of the optimal conditions (Scheme 1). The reaction color change from pale yellow to reddish pink was the first indicator used to investigate the success of the Ph-AuNP synthesis. The optical absorbance of the Ph-AuNPs was measured using UV-VIS spectroscopy over a wavelength range of 200–800 nm. To determine the shape and size of the Ph-AuNPs, the samples were examined using transmission electron microscopy (TEM; JEM-1400Flash, Jeol, Tokyo, Japan at 120 kV) and SEM (JSM-IT500HR, Jeol, Tokyo, Japan, at 15 kV). Ph-AuNP samples were prepared for TEM and SEM according to our previously published protocols [43]. The diameters of 100 Ph-AuNPs were measured by analyzing TEM micrographs using ImageJ 1.52a software and applying frequency distribution analysis using GraphPad Prism 10.2.3 software. To determine the elemental composition and distribution of the Ph-AuNPs, EDX and mapping analyses were performed using an EDX detector (STD-PC80, Jeol, Tokyo, Japan) attached to an SEM at 15 kV. The hydrodynamic diameter and zeta potential of the Ph-AuNPs were measured using a zetasizer (Malvern, UK). The functional groups surrounding the Ph-AuNPs were detected using a Fourier transform infrared instrument (Shimadzu, Kyoto, Japan) over a wavenumber of 400–4000 cm^−1^.

## 3. In Vitro Study

### 3.1. Cell Culture

SAOS-2, MG-63, and Vero cells were obtained from Nawah Co. (Cairo, Egypt). The cells were cultured in McCoy’s medium and Dulbecco’s Modified Eagle Medium media supplemented with 100 mg/mL of streptomycin, 100 units/mL of penicillin, and 10% fetal bovine serum. The cells were kept in a humidified environment under 5% CO_2_ at 37 °C.

### 3.2. Sulforhodamine B (SRB) Essay

The sulforhodamine B (SRB) assay was used to assess the viability of the SAOS-2, MG-63, and Vero cells before and after treatment with the Ph-AuNPs. Initially, 100 μL of cell suspension (5000 cells/well) was placed in 96-well plates and allowed to grow in complete media for 24 h. The cells were cultured in a humidified environment with 5% CO_2_ at 37 °C. Subsequently, the cells were treated with another 100 μL of medium containing Ph-AuNPs at various concentrations. A mixture of 1 mg of Ph-AuNPs and 1 mL of medium was sonicated for 15 min and sterilized using a UV lamp before being added to the cells. It is noteworthy that various concentrations (200, 100, 50, 25, 12.5, 6.25, 3.12, 1.56, 0.78, and 0.38) of chemically synthesized AuNPs (4–20 nm, nanotech, Egypt) were used as a positive control against the SAOS-2, MG-63, and Vero cells under the same experimental conditions. Following 24 h of drug exposure, approximately 150 μL of 10% trichloroacetic acid (TCA) was added for fixation after removing the media and incubated for 1 h at 4 °C. The TCA solution was then removed from the wells, and the cells were washed at least five times with distilled water. In total, 70 μL of freshly prepared SRB solution (0.4% *w*/*v*) was added, and the plates were kept at room temperature in a dark place for 10 min. Subsequently, each well was washed with 1% acetic acid. After air-drying overnight, the protein-bound SRB stain was solubilized with 150 μL of TRIS (10 mM) in each well, and the optical absorbance was measured at 540 nm using a FLUOstar Omega microplate reader (BMG Labtech, Ortenberg, Germany) [44].

### 3.3. Apoptotic Analysis Using Annexin VI and Propidium Iodide (PI)

An Annexin V-FITC apoptosis detection kit (Abcam, Cambridge, UK) combined with a two-channel flow cytometry system was used to determine the apoptotic and necrotic cell populations. MG-63 and SAOS-2 cells were treated with Ph-AuNPs at concentrations of 298.5 and 15.6 µg/mL, respectively, for 24 h. After treatment, 1 × 10^5^ cells were trypsinized and washed twice with ice-cold phosphate buffer saline (PBS) at pH 7.4. Subsequently, the cells were incubated in the dark with 500 µL of Annexin V-FITC/PI solution for 30 min at 25 °C, as guided by the manufacturer. After staining, the cells were analyzed using an ACEA Novocyte™ flow cytometer (ACEA Biosciences, San Diego, CA, USA), examining the FITC and PI fluorescent signals with FL1 and FL2 signal detectors, respectively (λex/em 488/530 nm for FITC and λex/em 535/617 nm for PI). We performed the acquisition of 12,000 events for all samples, and quadrant analysis was employed to quantify the FITC- and/or PI-positive cells. The results were calculated with ACEA NovoExpress™ 1.1.0 software (ACEA Biosciences, Santa Clara, CA, USA) [45].

### 3.4. Cell Cycle Arrest

MG-63 and SAOS-2 cells (1 × 10^5^ cells/well) were treated with IC_50_ concentrations of Ph-AuNPs at 298.5 and 15.6 µg/mL, respectively, for 24 h. After the cells were harvested by trypsinization, they were washed twice with 1 mL of ice-cold PBS at pH 7.4. Ice-cold ethanol (60%, 2 mL) was used to resuspend and fix the cells by incubation at 4 °C for 1 h. After that, the cells were washed twice again utilizing PBS at pH 7.4 and resuspended in 1 mL of PBS consisting of a mix of RNAase A and propidium iodide (PI) (50 μg/mL and 10 μg/mL, respectively). Following 20 min of incubation at 37 °C in the dark, the cells were subjected to flow cytometry analysis utilizing an FL2 (λex/em 535/617 nm) signal detector (ACEA Novocyte™ flow-cytometer, ACEA Biosciences) to determine the cellular DNA contents. A total of 12,000 events were obtained for all samples, and cell cycle distribution was analyzed using ACEA NovoExpress™ 1.1.0 software (ACEA Biosciences) [45].

### 3.5. Statistical Analysis

GraphPad Prism v. 10.2.3 (San Diego, CA, USA), Origin 8 (OriginLab Corporation, Northampton, MA, USA), and ImageJ (National Institutes of Health, Bethesda, MD, USA) software were used to analyze the data in the current study. The data are presented as means ± standard error of the mean (SEM), and statistical significance was detected using one-way analysis of variance (ANOVA). The data are considered to be statistically significant at a *p* < 0.05.

## 4. Results and Discussion

### 4.1. P. communis Strain AB_11_10 Identification

**Figure 1:**
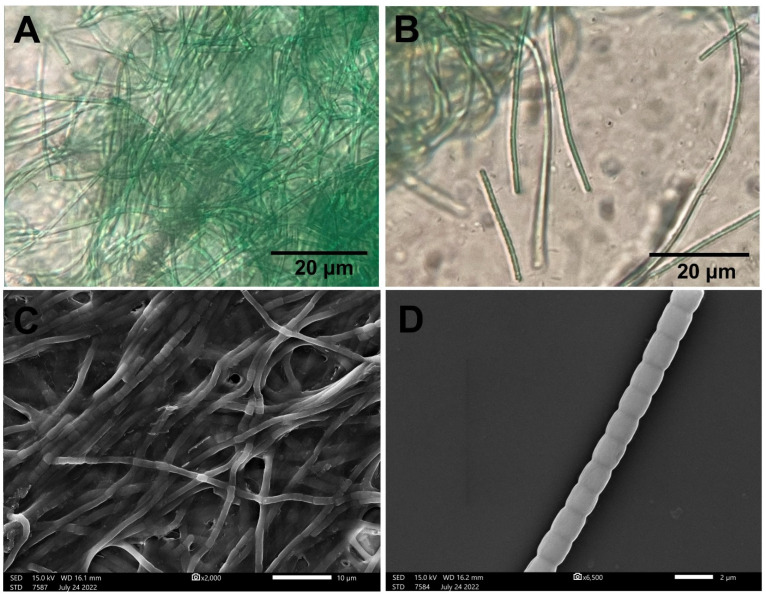
Illustrating the morphological appearance of *P. communis* strain AB_11_10 using light (**A**,**B**) and SEM (**C**,**D**). The *P. communis* strain AB_11_10 has segmented, pale blue-green-colored trichomes surrounded by a thin colorless sheath with rounded apical cells. Scale bar = 20 µm (**A**,**B**), 10 µm (**C**), and 2 µm (**D**).

**Figure 2:**
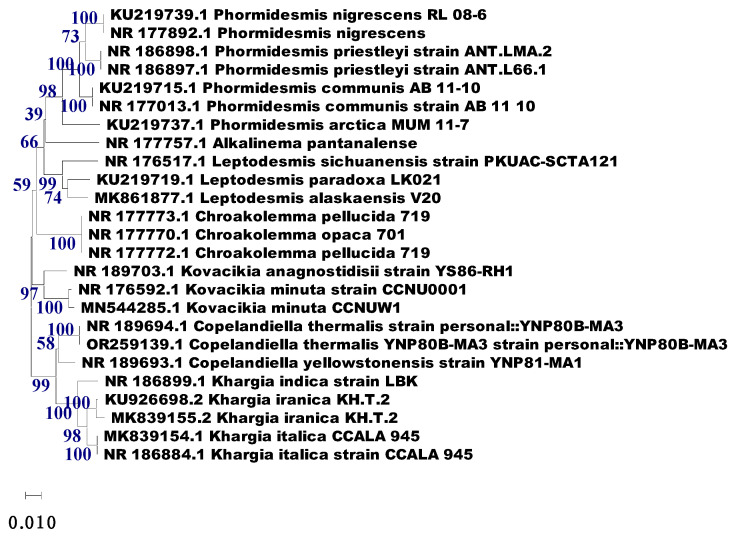
The phylogenetic tree of the 16s rRNA sequence of *P. communis* strain AB_11_10, built using the cluster method and MEGA4 software v.10.2.6.

The morphology of the isolated strain was identified using light microscopy and SEM. The isolate was segmented into filaments with a blue-green color and assembled into mats. The filaments were distinguished based on their apical ends (Figure 1). Free oscillatory movements were detected. The genomic DNA was isolated and purified to genetically identify the isolated strain, and 16s rRNA sequencing was performed. The data showed that the current strain was 98.0% identical to *Phormidesmis communis* strain AB_11_10 (Figure 2). Our strain was recorded in GenBank under the accession number OR790649. Davydov et al. morphologically identified *P. communis* as bright, pale blue-green, or olive-green trichomes surrounded by a thin colorless sheath; the width of these trichomes was 1.5–3.6 µm—the trichomes were composed of shorter cells (1.0–4.3 µm) with constrictions at cross-walls. The apical cells were rounded without calyptra [46].

### 4.2. Chemical Compositions of P. communis Strain AB_11_10 Algal Extract

LC-QTOF-MS was performed in positive and negative modes to detect the biomolecules and metabolites of *P. communis* strain AB_11_10. LC-QTOF-MS revealed 18 molecules that could be classified as anhydride, such as phthalic anhydride; organic compounds (aromatic, hydrocarbon, and heterocyclic compounds) such as O-xylene, pyridine, benzenesulfonic acid, dimethyl-, ptychantol A, (Z,6R)-N-(2,5-dimethylhex-5-enyl)-8-methylsulfanyl-6-propyldec-8-en-2-amine, and 1,6,9-Trioxa-3,12-dithiacyclotridecane; amino acids such as phenylalanine; esters such as dibutyl phthalate; phenolic compounds such as methyl eugenol; cyclic amides such as caprolactam; anionic surfactants such as dodecyl sulfate; vinyl compounds such as 4-vinylpyridine; and fatty acids such as 2-(4-hydroxyphenyl)ethylhentriacontanoate and linolenic acid (Table 1). Phthalic anhydride, O-xylene, caprolactam, 2-(4-hydroxyphenyl)ethylhentriacontanoate, linolenic acid, 4-vinylpyridine, ptychantol A, pyridine, dibutyl phthalate, and (Z,6R)-N-(2,5-dimethylhex-5-enyl)-8-methylsulfanyl-6-propyldec-8-en-2-amine had higher-intensity peaks than the other molecules [47].

### 4.3. AuNP Synthesis Using P. communis Strain AB_11_10

**Figure 3:**
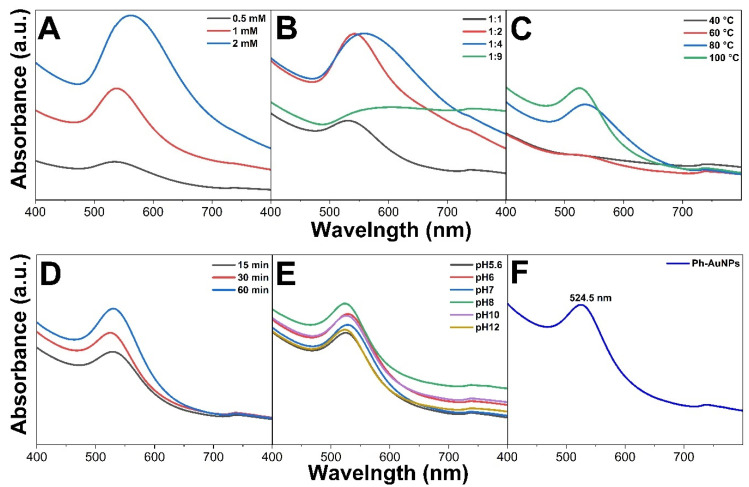
UV-VIS spectroscopy of Ph-AuNPs at various reaction conditions, (**A**) represents the influence of HAuCl_4_ concentrations (0.5, 1, and 2 mM) on the synthesis (size and stability) of Ph@AuNPs at other constant parameters, including algal extract/precursor ratio (1:1 *v*/*v*), temperature (80 °C), reaction time (30 min), and initial pH 5.6. (**B**) Represents influence of ratios (1:1, 1:2, 1:4, and 1:9) on the synthesis of Ph@AuNPs at constant 0.5 mM HAuCl_4_, 80 °C, 30 min, and pH 5.6. (**C**) Represents the influence of temperature (40, 60, 80, and 100 °C) on the synthesis of Ph@AuNPs at constant 0.5 mM HAuCl_4_, 1:1, 30 min, and pH 5.6. (**D**) Represents influence of reaction time (15, 30, and 60 min) on the synthesis of Ph@AuNPs at constant 0.5 mM HAuCl_4_, 1:1, 100 °C, and pH 5.6. (**E**) Represents influence of pH (5.6, 6, 7, 8, 10, and 12) on the synthesis of Ph@AuNPs at constant 0.5 mM HAuCl_4_, 1:1, 100 °C, and 30 min. (**F**) The absorption beak of Ph@AuNPs after being synthesized at optimized reaction conditions including 0.5 mM HAuCl_4_, 1:1 (*v*/*v*), 100 °C for 30 min, and pH 5.6.

The optimization of the synthesis of metallic NPs by physical and chemical conditions plays a critical role in increasing the yield and stability and determining the dimensions and geometries of NPs [48,49]. Cell culture conditions, such as metal salt concentrations, medium constituents, biomass yield, filtrate volume, and physical conditions, including light intensity, pH, time, and temperature, have been observed to affect NP size, synthesis rate, shape, and maximal yield [48,49]. To synthesize small, stable, and biocompatible Ph-AuNPs using an eco-friendly synthesis approach, a HAuCl_4_ solution was incubated with *P. communis* strain AB_11_10 algal extract, and the reaction conditions were varied to obtain the optimum conditions for synthesis. The results showed that an increment in the concentration from 0.5 (533.0 nm) to 1 (538.0 nm) to 2 mM (562.0 nm) caused a red shift in the Ph-AuNP wavelengths. These shifts indicated the formation of NPs with large sizes or aggregates of NPs. An increase in the precursor concentration against a constant reductant (algal extract) volume may affect the synthesis process of NPs by slowing the nucleation step, resulting in the formation of larger particles [50]. Similarly, HAuCl_4_/algal extract ratios (V_mL_/V_mL_) of 1:1 (533.0 nm), 1:2 (542.5 nm), 1:4 (559.2 nm), and 1:9 (600.5 nm) produced larger NPs and consequently enhanced the formation of NP aggregates [50]. Ph-AuNP synthesis was found to be temperature-dependent. The result showed that Ph-AuNPs did not form at 40 and 60 °C, whereas increasing the temperature to 80 (533.0 nm) or 100 °C (524.5 nm) caused a blue shift in the Ph-AuNP wavelengths. The optimum temperature to synthesize Ph-AuNPs was 100 °C. This can be attributed to the increase in temperature accelerating the nucleation reaction and enhancing the growth of small nanocrystals. Grigoris et al. observed that the size of AuNPs could be mitigated by controlling the synthesis temperature. The higher the temperature, the higher the rate of AuNP formation and the more uniform their size [51]. The duration of exposure to heat energy (reaction time) is a significant factor in Ph-AuNP synthesis. Increasing the reaction time from 15 (528.0 nm) to 30 (524.5 nm) to 60 min (529.3 nm) resulted in a blue shift in the wavelength of the Ph-AuNPs from 15 to 30 min, suggesting the formation of small NPs; however, increasing the reaction time to >30 min enhanced the formation of large particles. These data indicated that the complete synthesis of Ph-AuNPs occurred at 100 °C after 30 min. Changing the reaction pH is not desirable for the synthesis of Ph-AuNPs. In other words, Ph-AuNPs were successfully synthesized without adjusting the pH. The change in the initial pH from 5.6 (524.5 nm) to 6 (528.2 nm), 7 (528.1 nm), 8 (524.0 nm), 10 (526.0 nm), and 12 (524.3 nm) caused slight shifts in the Ph-AuNPs at pHs of 6, 7, and 10, or no change at pHs of 8 and 12. In summary, the optimum conditions for biofabricating Ph-AuNPs were as follows: 1 mL of 0.5 mM of HAuCl_4_ mixed with 1 mL of algal extract (2 mg/mL) exposed to 100 °C for 30 min at pH 5.6. Our data agree with Princy et al., who synthesized AuNPs using *Padina tetrastromatica* at various concentrations (0.1, 0.2, 1, and 2 mM HAuCl_4_), HAuCl_4_/algal extract (100 mg/mL) ratios, pH values, and reaction times. The authors found that increasing the algal volume to >2 mL against a constant volume of 1 mL of HAuCl_4_ resulted in broad Surface Plasmon Resonance (SPR) bands, indicating the agglomeration of AuNPs. At lower concentrations (0.1 mM), the authors did not observe any SPR peaks, whereas increasing the concentration to 0.5 and 1 mM resulted in a blue shift in the AuNP wavelengths from 536 to 532 nm. However, increasing the concentration from 1 to 2 mM caused a red shift in the AuNP wavelength to 541 nm. The authors reported that an initial pH of 3.2 was suitable for synthesizing homogeneous spherical AuNPs, and any change in pH resulted in the formation of polydisperse AuNPs. Contrary to our findings, the authors found that increasing the temperature from ambient to 90 °C caused a red shift from 532 to 544 nm, suggesting the formation of AuNP clusters. This could be attributed to the differences in biomolecule compositions among organisms and their heat sensitivity. In our research, biomolecules from the *P. communis* strain AB_11_10 were used to synthesize AuNPs that exhibited a great stability and chemical reactivity at high temperatures, presenting a challenge for *Padina tetrastromatica*. In the same study, the scholar reported that, by increasing the reaction time from 5 min to 48 h, the SPR intensity increased with a blue shift to 532 nm, indicating the formation of small AuNPs [50]. Another advantage of our synthesis technique mediated by the *P. communis* strain AB_11_10 is the production of small AuNPs in a short reaction time of 30 min. Subbulakshmi et al. successfully synthesized AuNPs using *Gelidiella acerosa* by mixing 1 mM of HAuCl_4_·3H_2_O with 25 mL of algal broth, with the mixture being heated at 60 °C for 30 min. This reaction resulted in the formation of a ruby-red AuNP suspension. These AuNPs had an SPR peak at 538 nm, a spherical shape, and a nanosize ranging from 5 to 20 nm [52]. Kumar et al. synthesized AuNPs by mixing auric chloride with *Hansenula anomala* as the reducing agent and an amine-terminated polyamidoamine dendrimer as a stabilizing agent. The resulting AuNPs exhibited an SPR peak at 550 nm. These particles were uniformly distributed and nearly monodisperse, with an average diameter of 14 nm [53] (Figure 3). We may conclude that the *P. communis* strain AB_11_10 has a high reduction potential for synthesizing smaller AuNPs compared to the other microorganisms indicated, based on the comparison of their data with our results.

### 4.4. Physicochemical Synthesis of AuNPs Synthesized by P. communis Strain AB_11_10

**Figure 4:**
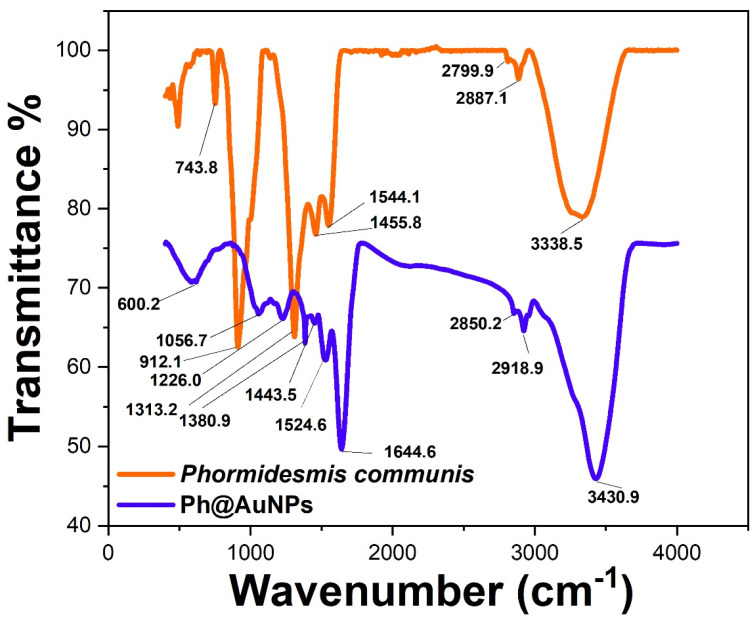
FTIR spectra of functional groups in *P. communis* strain AB_11_10 algal extract and surrounding the Ph-AuNPs.

To investigate the potential of *P. communis* strain AB_11_10 to reduce and stabilize the Ph-AuNPs and detect the functional groups emerging from the algal extract and coated Ph-AuNPs, we performed an FTIR analysis for both the *P. communis* strain AB_11_10 and Ph-AuNPs (Figure 4). The IR spectra of the *P. communis* strain AB_11_10 extract presented eight peaks at 3338.5, 2887.1, 2799.9, 1544.1, 1455.8, 1313.2, 912.1, and 743.8 cm^−1^, corresponding to the O-H stretching of alcohol, C-H stretching of alkane/aldhyde, N-H stretching of amine salt or C=C stretching of alkene, C-N stretching of amine, C-H bending of alkane, and C-H bending of alkane, respectively. The Ph-AuNPs presented 10 IR peaks at 3430.9, 2918.9, 2850.2, 1644.6, 1524.6, 1443.5, 1380.9, 1226.0, 1056.7, and 600.2 cm^−1^, relating to the O-H stretching of alcohol, C-H stretching of alkane/aldhyde, N-H stretching of amine salt or C=C stretching of alkene, C-N stretching of amine, C-H bending of alkane, O-H bending of phenol, C-O stretching of vinyl ether, C-O stretching of alcohol, and C-H bending of alkane. The IR peaks of the Ph-AuNPs were red-shifted compared to the *P. communis* strain AB_11_10 extract, suggesting the formation and conjugation of these functional groups on the NP surface. Moreover, the presence of O-H, N-H, C-H, C=C, N-O, and C-O on the Ph-AuNP surface suggests that the organic biomolecules of *P. communis* strain AB_11_10, such as peptides, polysaccharides, and fatty acids, could be the main reductants and stabilizing agents during the biofabrication of Ph-AuNPs. Based on the LC-QTOF data, we conclude that some of the 18 biomolecules, especially those that existed at high intensities, may have acted as reducing and stabilizing agents. To synthesize AuNPs, the precursor HAuCl_4_ solution was reduced by reducing agents which donated electrons to gold ions (Au^3+^), resulting in the formation of natural atoms of Au (nuclei) as an initial phase of the nucleation step; then, nuclei underwent a growth phase to form NPs. Stabilizing agents are very significant to prevent NPs from agglomeration and oxidation again. Caprolactam and phenylalanine in the *P. communis* strain AB_11_10 algal extract could be some of the reducing agents, whereas biomolecules such as linolenic acid work as stabilizing agents during AuNPs’ synthesis. More studies are needed to determine the exact molecules surrounding the AuNPs that are responsible for their reduction and stabilization. Our data agree with Asif et al., who synthesized copper oxide NPs using *Phormidium* sp. and reported the presence of O-H (3388 cm^−1^), C-H (2969 cm^−1^), N-H (1657 and 1536 cm^−1^), C-N (1452 cm^−1^), -COO (1402 cm^−1^), and C-O (1089 cm^−1^) [47]. González-Ballesteros et al. synthesized AuNPs using *Chondrus crispus*, *Gelidium corneum*, and *Porphyra linearis* extracts, analyzed the functional groups surrounding these AuNPs using FTIR, and compared the IR data with a GC analysis of algal extracts and other IR reference data. The authors concluded that polysaccharides may act as reducing and stabilizing agents, binding Au through sulfur atoms [54].

**Figure 5:**
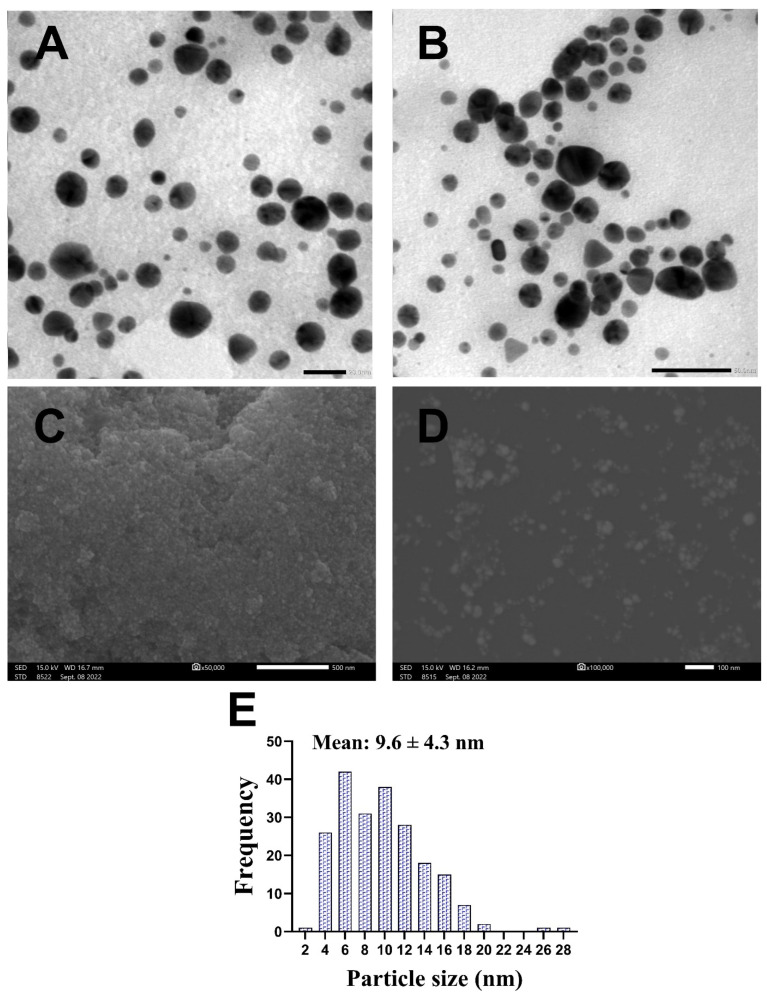
TEM (**A**,**B**) and SEM (**C**,**D**) imaging of Ph-AuNPs, revealing their quasi-spherical to triangular shapes, and (**E**) frequency distribution histogram of Ph-AuNPs, exhibiting their average diameter and nanosize range. Scale bars of 20 nm (**A**), 50 nm (**B**), 500 nm (**C**), and 100 nm (**E**).

The morphology of the Ph-AuNPs was examined using TEM and SEM (Figure 5). The data showed that the Ph-AuNPs were uniformly distributed with no agglomeration and had quasi-spherical, triangular, and rectangular shapes. These particles had a diameter of 9.7 ± 4.3 nm and a nano range from 2 to 28 nm. Our data agree with Mubarak Ali, who synthesized AuNPs using *Phormidium* sp. These AuNPs had triangular and hexagonal shapes with a size of 25 nm [37]. However, the average diameter of the Ph@AuNPs was smaller than that synthesized by *Phormidium* sp. Parial et al. bioreduced HAuCl_4_ to AuNPs using *P. valderianum* and *P. tenueand*. The authors reported that *P. valderianum* synthesized AuNPs at natural and basic pHs (7 and 9) with SPR peaks at 525 nm, whereas at pH 5, two absorption bands were recorded at 520 and 670 nm. The AuNPs formed at pH 5 were spherical (15 nm) and nanorods (411 × 32 nm), while those formed at pH 7 were spherical (8–17 nm) and triangular (24 nm). At pH 9, the AuNPs formed by *P. valderianum* were spherical (14 nm) and hexagonal (25 nm) in shape. The AuNPs synthesized by *P. tenue* showed maximum absorption peaks at 529 nm (pH 5 and 7) and 532 nm (pH 9). These AuNPs had spherical and irregular shapes (15 nm) [55]. Based on the previous data, we can conclude that *Phormidium* species have the potential to reduce the Au precursor to AuNPs with dominant shapes, including spherical, triangular, and hexagonal ones. Our data concur with Lenartowicz et al.’s findings, showing that triangular-shaped AuNPs can be obtained at 0.5 mM HAuCl_4_. The authors synthesized AuNPs intracellularly using *Anabaena laxa* at various concentrations of HAuCl_4_ (0.1, 0.5, and 1 mM). The authors observed that the resulting AuNPs had various shapes, which varied according to the initial HAuCl_4_ concentration. For instance, small and spherical AuNPs (3–5 nm) were formed at 0.1 mM, whereas triangular, hexagonal, and irregular shapes were formed at 0.5 and 1 mM. The size of the AuNPs formed at 0.5 mM ranged from 0 to 30 nm; however, the larger NPs started from 30 to 100 nm, while the size of the AuNPs at 1 mM ranged from 20 to 50 nm, with the larger particle size being >100 nm [56].

**Figure 6:**
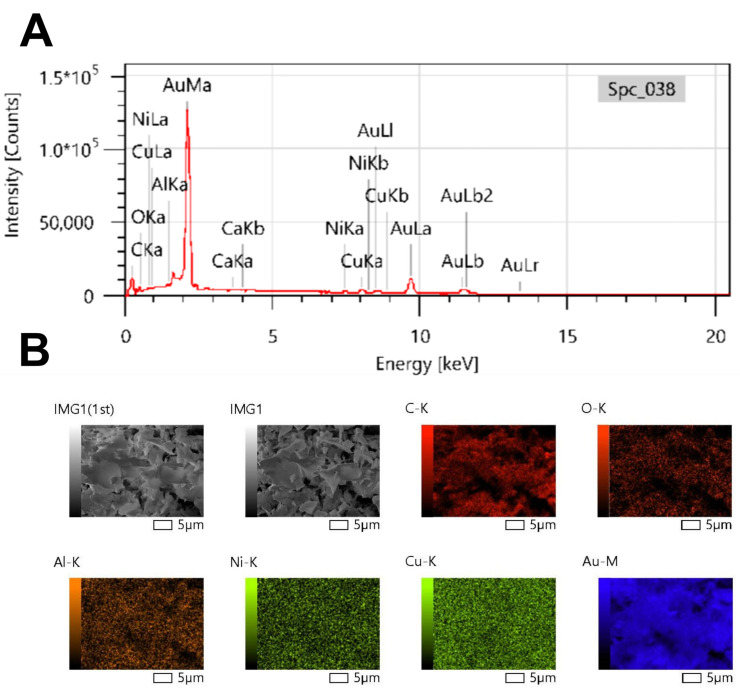
Energy diffractive X-ray analysis (**A**) and mapping analysis (**B**) of Ph-AuNPs reveal their elemental composition and distribution.

**Figure 7:**
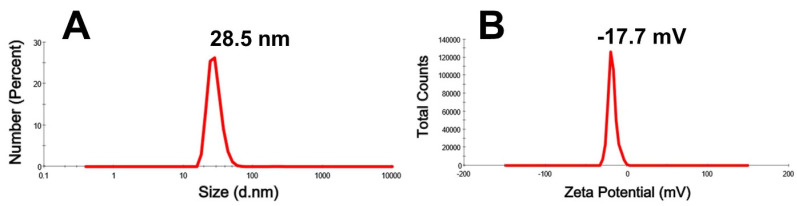
Illustrating the hydrodynamic diameters (HDs) of Ph-AuNPs (**A**,**B**) and their potential charge.

To investigate the elemental composition and distribution of the Ph-AuNPs, we performed EDX and mapping analyses (Figure 6 and Table 2). The data revealed that the main elements in the nanostructured Ph-AuNPs were Au metal (76.10 ± 3.14%), followed by carbon (17.90 ± 3.36%), copper (2.85 ± 0.85%), and oxygen (2.37 ± 1.04%), suggesting that the *P. communis* strain AB_11_10 successfully synthesized and coated the Ph-AuNPs. Trace amounts of other elements, including calcium, aluminum, and nickel, were also detected. These elements may be related to algal nutrients [57]. The hydrodynamic diameters (HDs) of the Ph-AuNPs in the aqueous phase were 28.5 nm, while their charge was −17.7 mV (Figure 7). The negativity of the Ph-AuNPs may have been due to the presence of negatively charged functional groups such as C-O.

### 4.5. Anticancer Activity of AuNPs Synthesized by P. communis Strain AB_11_10

**Figure 8:**
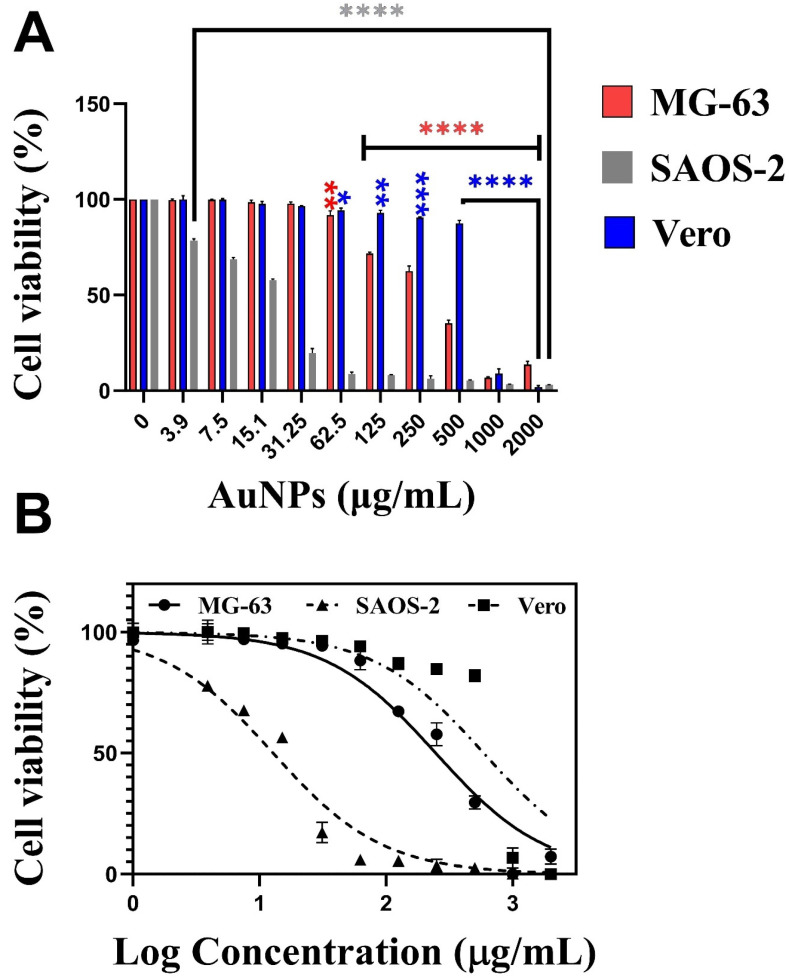
Antiproliferative activity of Ph-AuNPs against two osteosarcoma cancer cell lines (MG-63 and SAOS-2) and normal kidney (Vero) cells represented as a bar chart (**A**) and sigmoidal graph for IC_50_ detection (**B**). **** *p* < 0.0001, *** *p* < 0.0001, ** *p* < 0.005, and * *p* < 0.01.

To investigate the cytotoxicity and biocompatibility of the Ph-AuNPs synthesized by *P. communis* strain AB_11_10, MG-63, SAOS-2, and Vero cell lines were exposed to different concentrations of Ph-AuNPs (2000, 1000, 500, 250, 125, 62.5, 31.25, 15.1, 7.5, and 3.9 µg/mL). A total of 50% inhibition in the cell viability of the MG-63, SAOS-2, and Vero cells was observed at 297.5, 15.5, and 861.4 µg/mL, respectively (Figure 8). These results show that Ph-AuNPs are biocompatible at lower concentrations compared to in normal kidney cells. Furthermore, these low concentrations of Ph-AuNPs were sufficient to reduce the viability of malignant MG-63 and SAOS-2 cells by >50%. However, the SASO-2 cells were more sensitive to the Ph-AuNPs than the MG-63 cells. This difference in the therapeutic responses of the MG-63 and SAOS-2 cells could be attributed to the nature of malignant genetic and cell biology and/or the biological interfaces between the NP surface ligands (functional groups) and cell receptors. The cytotoxicity of the Ph-AuNPs was likely due to their potential to induce apoptosis via enhancing the formation of ROS, consequently causing oxidative stress [58]. On the other hand, chem@AuNPs suppressed 50% of the MG-63, SAOS-2, and Vero cell viability at an IC_50_ of 72, 62, and 35 µg/mL, respectively (Figure S1). These data suggest that biogenic Ph@AuNPs may be more biocompatible with kidney cell lines, showing greater potency and selectivity against SAOS-2 cells compared to citrate-coated AuNPs. Chem@AuNPs are more effective against MG-63 cells than Ph@AuNPs. However, the toxicity of Chem@AuNPs at the IC50 of MG-63 cells exceeded the safe level for Vero cells. The effectiveness of the Ph@AuNPs may be attributed to the presence of an algal corona that coated the Ph@AuNPs, enhancing their stability in a cellular context and preventing their agglomeration, thereby maintaining their effectiveness. Additionally, this algal corona may improve the connections between the nanoparticle surface and cell receptors.

Steckiewicz et al. assessed the toxicity of various AuNPs, including rods, spheres, and stars, against MG-63, hFOB 1.19, and 143 B cells. The authors found that both rod-shaped and star-shaped AuNPs were toxic to all the cell types, whereas spherical AuNPs did not exhibit any toxicity. The authors suggested that the cytotoxicity and anticancer activities of AuNPs are shape-dependent [25]. Another study by Rahim et al. showed that AuNPs capped with glycation products (a spherical shape with an average diameter of 24.3 nm) exhibited antiproliferative activity against SAOS-2 cell lines [26].

**Figure 9:**
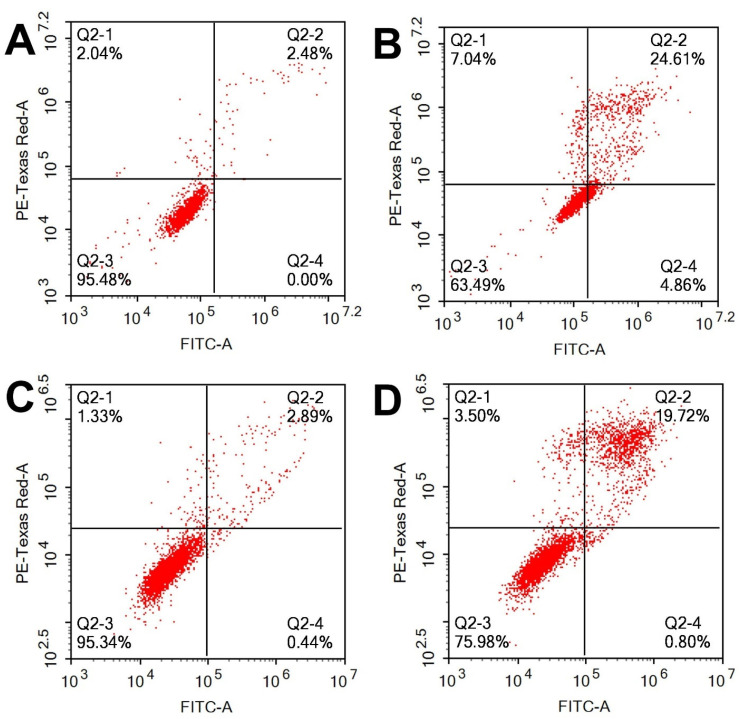
Flow cytometric analysis of apoptosis using Annexin V/PI staining. Untreated and treated MG-63 cells (**A**,**B**) with 297.5 µg/mL Ph-AuNPs and untreated and treated SAOS-2 cells (**C**,**D**) with 15.5 µg/mL Ph-AuNPs for 24 h.

**Figure 10:**
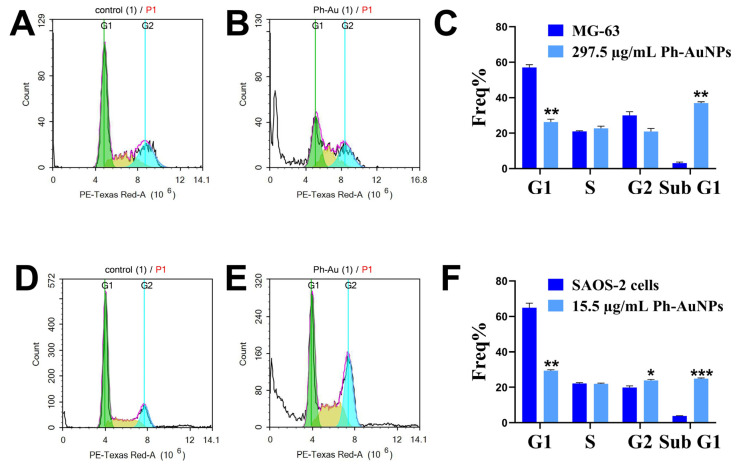
Influence of Ph-AuNPs on cell cycle of MG-63 and SAOS-2 cell lines. Untreated (**A**) and treated MG-63 cells with Ph-AuNPs (**B**) and their frequency (Freq) % of cell cycle phases (**C**). Untreated (**D**) and treated SAOS-2 cells with Ph-AuNPs (**E**) and their frequency (Freq) % of cell cycle phases (**F**). The star refers to the significance based on the paired *t*-test in which * *p* < 0.03, ** *p* < 0.005, and *** *p* < 0.0003.

To understand how Ph-AuNPs might enhance the apoptotic pathway, we performed an annexin/PI double staining analysis (Figure 9). The scatter plots of the treated and untreated MG-63 and SAOS-2 cells have four quadrants: Q2-3 represents viable cells (annexin − ve, PI − ve), Q2-4 represents early apoptotic cells (annexin + ve, PI − ve), Q2-2 represents late apoptotic cells (annexin + ve, PI + ve), and Q2-1 indicates necrotic cells (PI +ve) (Figure 9). MG-63 cells exposed to 297.5 µg/mL of Ph-AuNPs for 24 h exhibited four-quadrant plots different from untreated MG-63 cells. In total, there were 63.4% viable cells; 4.86% of the treated MG-63 cells were in the early apoptotic phase, 24.61% of the cells were in the late apoptotic phase, and 7.07% had undergone necrosis. SAOS-2 cells treated with 15.6 µg/mL of Ph-AuNPs for 24 h revealed 75.98% of viable cells, 0.80% in the early apoptotic phase, 19.72% in the late apoptotic phase, and 3.50% that had undergone necrosis. These results demonstrate the potential of Ph-AuNPs to enhance programmed cell death in both MG-63 and SAOS-2 cell lines [58]. To investigate the role of Ph-AuNPs in enhancing cell cycle arrest, untreated MG-63 and SAOS-2 cells treated with Ph-AuNPs for 24 h were stained with PI, and the cell populations in each phase were estimated using flow cytometry (Figure 10). The MG-63 and SAOS-2 cells treated with Ph-AuNPs showed an increase in their subpopulation in sub-G1 (35.79 and 24.68%, respectively) compared to the untreated cells (4.63 and 3.61%, respectively). This sub-G1 cell population is believed to be the outcome of endonuclease activation and subsequent DNA leakage from cells, indicating that a high proportion of the population undergoes apoptosis. This increment in the sub-G1 population consequently correlated with a reduction in the G1 populations of the MG-63 and SAOS-2 cells (29.37 and 30.23%, respectively) after treatment with the Ph-AuNPs compared to the untreated cells (60.09 and 63.73%, respectively). This reduction in the G1 population could indicate that a portion of the population was arrested in the S or G2/M phases due to DNA damage or cellular stress. In the S phase, the percentage of the MG-63 and SAOS-2 cell populations treated with Ph-AuNPs decreased slightly compared to that in the untreated cells. Furthermore, the percentage of the SAOS-2 cell population in the G2 phase was increased compared to the untreated cells, indicating that the Ph-AuNPs arrested the SAOS-2 cell line in the G2/M phase, whereas the percentage of the MG-63 population in the G2 phase was reduced after treatment compared to the untreated cells, suggesting that a portion of the population was arrested in the G1 or S phases due to DNA damage, disrupted cell cycle regulation, or cellular stress. Additionally, this could be referred to as mitotic arrest, in which cells are unable to progress past the G2 phase to the mitotic phase [59]. These data suggest that the Ph-AuNPs acted as potent antiproliferative and anti-apoptotic agents that significantly enhanced cell cycle arrest.

## 5. Conclusions

Here, we assessed, for the first time, the potential of *P. communis* strain AB_11_10 to synthesize Ph-AuNPs and demonstrated their lethal activity against osteosarcoma cancer cells. It was found that exposing HAuCl_4_ to the *P. communis* strain AB_11_10 algal extract under the optimum reaction conditions resulted in the formation of small, well-dispersed Ph-AuNPs. The optimized parameters for the Ph-AuNPs’ synthesis were 0.5 mM of HAuCl_4_, 1:2 ratio (*v*/*v*) HAuCl_4_/algal extract, and 100 °C for 30 min at pH 5.6. The Ph-AuNPs had an SPR at 524.5 nm, quasi-spherical to triangular shapes, and an average diameter of 8.8 ± 0.3 nm. Au represented 76.10 ± 3.14% of the Ph-AuNP sample mass, and elements such as carbon and oxygen were also detected, indicating that *P. communis* strain AB_11_10 successfully synthesized Ph-AuNPs. The diameter of the Ph-AuNPs in the aqueous system was 28.5 nm, whereas their potential charge was −17.7. The IR spectra of the Ph-AuNPs revealed that algal organic compounds, amino acids, and fatty acids were the main reductants and stabilizing substances in the Ph-AuNP synthesis process. The Ph-AuNPs demonstrated potent anticancer activity against osteosarcoma cell lines by enhancing apoptosis and arresting the cell cycle. In summary, the current investigation showed that the *P. communis* strain AB_11_10 represents a novel, sustainable, eco-friendly, and inexpensive natural biomachinery for the biosynthesis of small and stable AuNPs with potent therapeutic activity and selectivity against malignant cells.

## Figures and Tables

**Table 1:** LC-QTOF-MS analysis of phytochemicals of *P. communis* strain AB_11_10 algal extract.

No	RT	−/+	Formula	Precursor Mass	Found at Mass	Compound
1	1.92	+	C_8_H_4_O_3_	149.023	149.0234	Phthalic anhydride
2	2.11	−	CH_4_O_8_S	174.957	174.9560	Hydroxy trihydroxymethyl sulfate
3	2.26	+	C_8_H_4_O_3_	149.023	149.0233	Phthalic anhydride
4	11.43	+	C_8_H_10_	107.085	107.0852	O-xylene
5	10.5	−	C_9_H_11_NO_2_	164.072	164.0713	Phenylalanine
6	11.96	+	C_16_H_22_O_4_	279.172	279.1594	Dibutyl phthalate
7	14.06	+	C_11_H_14_O_2_	179.107	179.1063	Methyl eugenol
8	14.23	+	C_6_H_11_NO	114.091	114.0909	Caprolactam
9	15.78	+	C_5_H_5_N	80.049	80.0492	Pyridine
10	17.04	−	C_8_H_10_O_3_S	185.119	185.1177	Benzenesulfonic acid, dimethyl-
11	19.44	−	C_39_H_70_O_3_	585.526	585.5253	2-(4-Hydroxyphenyl)ethylhentriacontanoate
12	19.68	−	C_12_H_26_O_4_S	265.18	265.1465	Dodecyl sulfate
13	19.84	+	C_8_H_4_O_3_	149.023	149.0231	Phthalic anhydride
14	20.29	+	C_8_H_16_O_3_S_2_	225.061	225.0613	1,6,9-Trioxa-3,12-dithiacyclotridecane
15	21.25	+	C_22_H_43_NS	354.319	354.3191	(Z,6R)-N-(2,5-dimethylhex-5-enyl)-8-methylsulfanyl-6-propyldec-8-en-2-amine
16	21.63	+	C_28_H_22_O_4_	423.160	423.1590	ptychantol A
17	22.33	+	C_7_H_7_N	106.065	106.0653	4-Vinylpyridine
18	23.36	−	C_18_H_30_O_2_	577.422	577.4247	Linolenic acid

**Table 2:** The elemental composition of Ph-AuNPs.

Element	Line	Mass%	Atom%
C	K	17.90 ± 3.36	71.37 ± 0.37
O	K	2.37 ± 1.04	7.09 ± 0.09
Al	K	0.21 ± 0.25	0.37 ± 0.01
Ca	K	0.16 ± 0.30	0.19 ± 0.01
Ni	K	0.41 ± 0.47	0.34 ± 0.01
Cu	K	2.85 ± 0.85	2.15 ± 0.02
Au	M	76.10 ± 3.14	18.50 ± 0.02
Total		100.00	100.00

## Data Availability

Additional data to those presented here are available from the corresponding author upon reasonable request.

## Supplementary Materials

The following supporting information can be downloaded at: https://www.mdpi.com/article/10.3390/biomedicines12071570/s1, Figure S1: Antiproliferative activity of Chem@AuNPs against two osteosarcoma cancer cell lines (MG-63 and SAOS-2) and normal kidney (Vero) cells represented as a bar chart (A) and sigmoidal graph for IC50 detection (B). **** *p* < 0.0001, *** *p* < 0.0001, and ** *p* < 0.005.
