# Supplementary material for: Apoptotic Induction by Biosynthesized Gold Nanoparticles Using Phormidesmis communis Strain AB_11_10 against Osteosarcoma Cancer
Source: Biomedicines. 2024 Jul 15;12(7):1570. doi: 10.3390/biomedicines12071570 (PMC11274524; doi:10.3390/biomedicines12071570)
Supplement: Supplementary file 1 [file biomedicines-12-01570-s001.zip › biomedicines-3051958-supplementary.pdf]

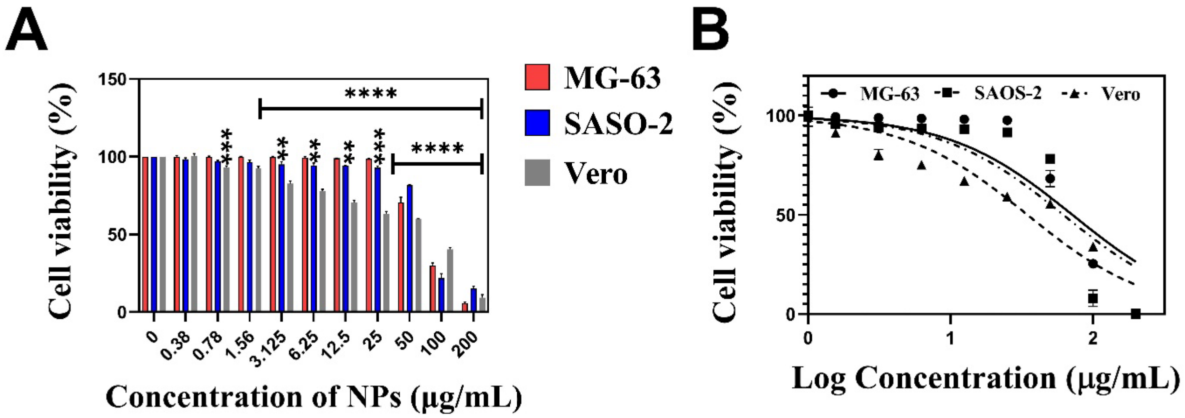

Figure S1: Antiproliferative activity of Chem@AuNPs against two osteosarcoma cancer cell lines (MG-63 and SAOS-2) and normal kidney (Vero) cells represented as a bar chart (A) and sigmoidal graph for IC50 detection (B).
**** $p < 0.0001$, *** $p < 0.0001$, and ** $p < 0.005$.
